# Exosomal microRNAs in cancer: Potential biomarkers and immunotherapeutic targets for immune checkpoint molecules

**DOI:** 10.3389/fgene.2023.1052731

**Published:** 2023-02-17

**Authors:** Faizah Alotaibi

**Affiliations:** College of Science and Health Professions, King Saud bin Abdulaziz University for Health Sciences and King Abdullah International Medical Research Center, Ministry of National Guard Health Affairs, Riyadh, Saudi Arabia

**Keywords:** exosomal miRNA, extracellular vesicles (EVs), cancer, small non-coding RNAs (sncRNAs), biomarker, clinical implication, liquid bioposy, immune checkpoint molecules

## Abstract

Exosomes are small extracellular vesicles with a lipid bilayer structure secreted from different cell types which can be found in various body fluids including blood, pleural fluid, saliva and urine. They carry different biomolecules including proteins, metabolites, and amino acids such as microRNAs which are small non-coding RNAs that regulate gene expression and promote cell-to-cell communication. One main function of the exosomal miRNAs (exomiRs) is their role in cancer pathogenesis. Alternation in exomiRs expression could indicate disease progression and can regulate cancer growth and facilitate drug response/resistance. It can also influence the tumour microenvironment by controlling important signaling that regulating immune checkpoint molecules leading to activation of T cell anti-tumour immunity. Therefore, they can be used as potential novel cancer biomarkers and innovative immunotherapeutic agents. This review highlights the use of exomiRs as potential reliable biomarkers for cancer diagnosis, treatment response and metastasis. Finally, discuses their potential as immunotherapeutic agents to regulate immune checkpoint molecules and promote T cell anti-tumour immunity.

## 1 Introduction

Extracellular vesicles (EVs) were initially described by Harding in 1983 (Harding et al., 1983) and later confirmed by Pan in 1985 (Pan et al., 1985). At first, they were known as vehicles for clearance of cellular “waste” which results from cell metabolism with no influence on neighboring cells. This concept, however, switched after the finding of other biomolecules, e.g., amino acids, fatty acid, and nucleic acids including small RNAs particularly microRNA in 2007 (Théry et al., 2002; Valadi et al., 2007). Upon release from cells, they can circulate to the neighboring cells and internalized *via* endocytosis (Larrea et al., 2016) and ultimately result in cell-to-cell communication and contribute to reprogram the recipient cells (Meldolesi, 2018). Thus, exosomal microRNAs (exomiRs) play a major role in intercellular communication to regulate gene expression (Théry, 2011; Kozomara and Griffiths-Jones, 2014). All type of cells including cancer cells can naturally secrete exosomes (Théry et al., 2002) in which can be isolated from different bio-fluids including urine and serum. This secretion is a result of cells undergoing difference condition such as apoptosis/necrosis or chronic inflammation which suggest a possible source of less-invasive method of the so-called “liquid biopsy”.

**FIGURE 1:**
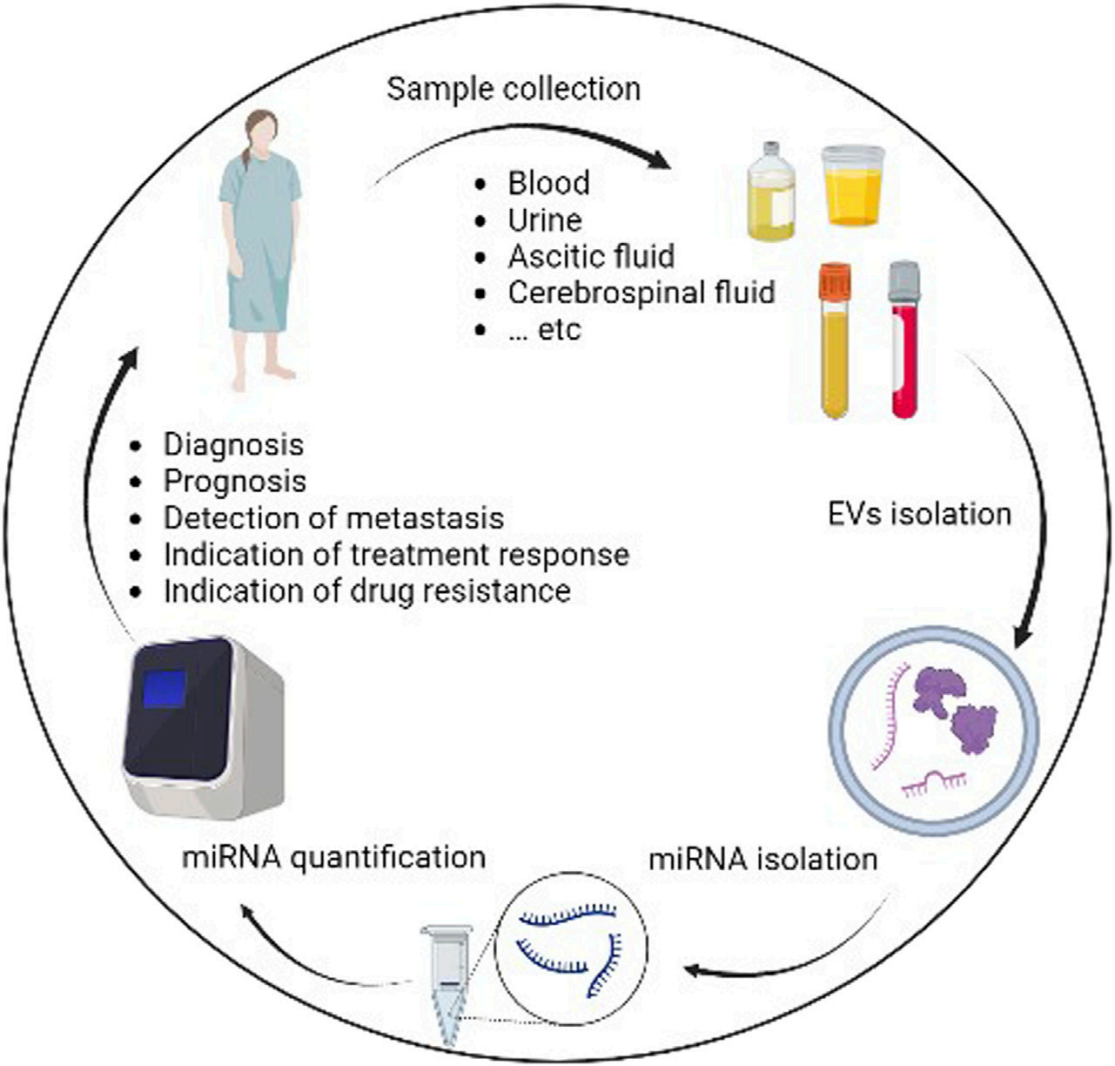
Workflow of processing and utilization of exomiRs for cancer clinical implication.

ExomiRs can be isolated from variety of body fluid including blood, saliva, urine, and breast milk (Figure 1) by differential ultracentrifugation (Amorim et al., 2017) which results in separation of exomiRs from contaminated cells, cellular debris and other EVs subtype such as apoptotic bodies and microparticles (Théry et al., 2006). Further techniques based on size exclusion such as chromatography and Optiprep™ density gradient have been used to increase the purity of exomiRs isolation (Boing et al., 2014; Lobb et al., 2015). In 2014, the International Society for EVs has published recommendations for EVs definition and their functions (Lotvall et al., 2014). Protein expression for at least three EV markers (e.g., CD63, CD81 and CD9) is typically used to identify the purity of the sample (Ekström et al., 2022). Furthermore, the use of two techniques (e.g., electron microscopy and nanoparticle tracking analysis) are recommended to illustrate the degree of heterogeneity in the sample (Dragovic et al., 2011; Van der Pol et al., 2014). To help standardize the protocols, EV-TRACK knowledgebase project (Can be access at http://evtrack.org) was developed and conducted by international collaboration from several countries (Van Deun et al., 2017). This helps researchers to add methodological parameters into central repository to quantifies their protocol. It is recommended to follow protocol based on the biofluid resources (Ramirez et al., 2018). For example, when working with blood as a source for the exomiRs, challenges like hemolysis has been shown to decrease the expression of some microRNAs and agitation used to stimulate blood transportation can lead to release of platelets (Ramirez et al., 2018). Another important factor is the storage time which can affect exomiRs yield (Ramirez et al., 2018). Comparison study shows increase amount of exomiRs isolated from the blood after storage for 3 h compared to freshly isolated blood which can be due to platetets-drived EVs (Ramirez et al., 2018).

In cancer, alternation in exomiRs expression is a well-known feature in cancer (Alotaibi, 2021). It can bind to messenger RNA which results in posttranscriptional gene regulation that promote cancer cell processes including progression and metastasis (Lin and Gregory, 2015). This process is critical for carcinogenesis, which help cancer cells thrive in the tumour microenvironment. For example, crosstalk between cancer cell and surrounding cells including immune cells and stromal cells leading to pre-metastatic niche development. Therefore, investigating exomiRs secreted from cancer cells is important to reveal cancer behavior and metastasis and understand their potential role as cancer biomarkers and to develop novel immunotherapeutic agents for cancer. This review describes the use of exomiRs as potential biomarkers for cancer diagnosis/prognosis, treatment response, and cancer metastasis and explore their role as innovative immunotherapeutic agents for cancer.

## 2 ExomiRs as novel biomarkers for cancer

Early treatment intervention for cancer can positively impact overall survival and result in desirable treatment outcome (Hiom, 2015). The standard method to confirm diagnosis is usually invasive biopsy of suspected tissue. Although is reliable, such a method can be difficult to perform due to tissue inaccessibility and possible damage to the normal tissue with the risk to stimulate metastasis (Shyamala et al., 2014). Therefore, using less invasive and effective method is important to improve early cancer diagnosis and predict treatment response and metastasis. Changes in levels of exomiRs can be noticed before the patients can develop clear symptoms for cancer and during cancer development (Chen et al., 2008a). Screening variant expression of tissue-specific exomiRs isolated from various body fluids are shown to prove the diagnosis and prognosis of different type of cancer (Table 1). Predict treatment response (Table 2) and metastasis (Table 3).

**TABLE 1:** List of exomiRs used for cancer diagnosis.


Centralize the miRNAs ID	Pattern of expression	Cancer type	Tumor stage	Body fluid source	References
ExomiR-92	Increased	Colorectal cancer	All stages	Plasma and tissue samples	Ng et al. (2009)
ExomiR-17-5p and exomiR-92a-3p	Increased	Colorectal cancer	Higher clinical stages	Serum	Fu et al. (2018)
ExomiR-17-5p, exomiR-21, exomiR-106a and exomiR-106b	Increased	Gastric cancer	Stages I-IV	Plasma	Tsujiura et al. (2010)
ExomiR-21, exomiR-155, exomiR-210 and exomiR-196a	Increased	Pancreatic adenocarcinoma patients	All stages	Plasma	Wang et al. (2009)
ExomiR-500	Increased	Hepatocellular carcinoma	Not mentioned	Serum	Yamamoto et al. (2009)
ExomiR-184	Increased	Squamous-cell carcinoma of the tongue	Not mentioned	Plasma	Wong et al. (2008)
ExomiR-125a and exomiR-200a	Decreased	Oral squamous-cell carcinoma	Stages I-IV	Saliva	Park et al. (2009)
63 exomiRs	Increased	Non-small-cell lung cancer	Stages I-IV	Serum	(Chen et al., 2008a)
34 exomiRs	Increased	Asymptomatic NSCLC	Early-stage nodule (Ia or Ib)	Serum	Bianchi et al. (2011)
21 exomiRs	Increased	Lung cancer	12–28 before and at the time of diagnosis	Plasma	Boeri et al. (2011)
hsa-miR-212, -214, −205, −210, −203, −191, −192, −146, −155, −21, −106a and -17-3p	Increased	Lung adenocarcinoma	Stages I-IV	Tissues biopsy	Rabinowits et al. (2009)
ExomiR-200-5p, exomiR-379, exomiR-139-5p and exomiR-378a	Increased	Lung adenocarcinoma	Early-stage nodule (Ia or Ib)	Tissue biopsy and plasma	Cazzoli et al. (2013)
ExomiR-141 and other 15 exomiRs	Increased	Prostate cancer	Stage 3 and 4	Serum	Lodes et al. (2009)
ExomiR-21-5p, exomiR-574-3p, and exomiR-141-5p	Increased	Prostate cancer	Not mentioned	Urine	Samsonov et al. (2016)
ExomiR-92, exomiR-93 and exomiR-126	Increased	Epithelial ovarian cancer	Stages I-IV	Serum	Resnick et al. (2009)
ExomiR-21, exomiR-141, exomiR-200a, exomiR-200c, exomiR-200b, exomiR-203, exomiR-205 and exomiR-214)	Increased	Ovarian cancer	Various stages	Serum	Taylor and Gercel-Taylor (2008)
ExomiR-195	Increased	Breast cancer	Stage IV	Serum, plasma, or whole blood	Heneghan et al. (2010)
ExomiR-101 and exomiR-372	Increased	Breast cancer and triple-negative breast cancer	pT1 pT2-4	Serum	Eichelser et al. (2014)
ExomiR-199a-3p	Increased	Pediatric neuroblastoma	Not mentioned	Plasma	Ma et al. (2019)
ExomiR-16	Increased	Pediatric acute lymphoblastic leukemia	Not mentioned	Blood	Kaddar et al. (2009)
ExomiR-21	Increased	Pediatric Hepatoblastoma	Not mentioned	Plasma	Liu et al. (2016b)
ExomiR-7112-5p, exomiR-885-3p and exomiR-1245a	Increased	Pediatric acute myeloid leukaemia	Risk of disease recurrence	Plasma	Zampini et al. (2017)
ExomiR-25-3p	Increased	Osteosarcoma	Not mentioned	Serum	Fujiwara et al. (2017)
ExomiR-125b	Decreased	Ewing’s sarcoma	Metastasis and non-metastasis	Serum	Nie et al. (2015)
ExomiR-21	Increased	Diffuse large B cell lymphoma	Stages I-IV	Serum	Lawrie et al. (2008)
ExomiR-92a	Decreased	Acute leukemias	Not mentioned	Plasma	Tanaka et al. (2009)
ExomiR-148a, exomiR-181a, exomiR-20a, exomiR-221, exomiR-625, and exomiR-9	Increased	Multiple myeloma	Not mentioned	Plasma	Huang et al. (2012a)
ExomiR-32, exomiR-98 and exomiR-374	Decreased	Chronic lymphocytic leukemia	Not mentioned	Blood	Rahimi et al. (2021)
ExomiR-451	Increased	Chronic myelogenous leukemia	Chronic stage	Plasma	Keramati et al. (2021)

**TABLE 2:** List of exomiRs associated with drug-response.

Centralize the miRNAs ID	Pattern of expression	Cancer type	References
ExomiR-181b	Increased	Better response to 5-fluorouracil in Colorectal cancer	Nakajima et al. (2006)
ExomiR-140 and ExomiR-215	Increased	Resistance to methotrexate, 5-fluorouacil, and Tomudex in human osteosarcoma and colon cancer cells	Song et al. (2009), Song et al. (2010)
ExomiR-19a	Increased	FOLFOX resistance in Advanced Colorectal Cancer Cases	Chen et al. (2013)
ExomiR-155	Increased	Gemcitabine Resistance in Pancreatic Ductal Adenocarcinoma	Mikamori et al. (2017)
ExomiR‐34a	Decreased Increased	Docetaxel resistance in castration-resistant prostate cancer Increased sensitivity to sorafenib in hepatocellular carcinoma cell lines	Corcoran et al. (2014) (Yang et al., 2014)
ExomiR-21	Increased	Cisplatin resistance in gastric cancer (In mice)	Zheng et al. (2017)
ExomiR-122	Decreased	Resistance to taxol in liver cancer	Sun et al. (2016)
ExomiR-128b	Loss of expression	Better response to gefitinib in non-small cell lung cancer	Weiss et al. (2008)
ExomiR-21	Increased	Resistant to docetaxel-based chemotherapy in prostate cancer	Zhang et al. (2011a)
ExomiR-9	Increased	Increase sensitivity of ovarian cancer to DNA damaging chemotherapy	Sun et al. (2013)
ExomiR‐125 b	Deletion	Better respond to chemotherapy with anthracycline in breast cancer	Climent et al. (2007)
ExomiR-195, ExomiR-455-3p, and ExomiR-10a miR-221	Increased Increased	Temozolomide resistance in glioma	Ujifuku et al. (2010) Yang et al. (2017a)
ExomiR-29a and exomiR-100	Increased	Drug resistance in pediatric acute promyelocytic leukemia	Zhang et al. (2011b)
ExomiR-142-3p and exomiR-17-92	Increased	Glucocorticoid-resistant B cell precursor acute lymphoblastic leukemia	Sakurai et al. (2019)
ExomiR-99a, exomiR-100, and exomiR-125b	Increased	Resistance to daunorubicin and vincristine in pediatric acute lymphoblastic leukemia	Schotte et al. (2011)
ExomiR-142-5p, exomiR-199b, exomiR-217, exomiR-221, and exomiR-365a-3p	Decreased	Tyrosine kinase inhibitors resistance in chronic myelogenous leukemia	Yeh et al. (2016), Jiang et al. (2018), Klümper et al. (2020)
ExomiR-145-3p and exomiR-155	Decreased	Bortezomib resistance in multiple myeloma	Amodio et al. (2019), Wu et al. (2020)
ExomiR-217	Increased	Sensitizes AML to doxorubicin	Xiao et al. (2017)
ExomiR-143	Increased	Enhances chemosensitivity of acute myeloid leukaemia to cytosine arabinoside	Zhang et al. (2020a)
ExomiR-181a/b	Increased	Fludarabine response in chronic lymphocytic leukemia	Zhu et al. (2012)

**TABLE 3:** List of exomiRs associated with tumour recurrence.

Centralize the miRNAs ID	Pattern of expression	Tumour type	References
ExomiR-106a-5p	Increased	Gastric cancer metastasis	Yuan et al. (2016)
ExomiR-4772-3p	Increased	Recurrence in stage II and III colon cancer	Liu et al. (2016a)
ExomiR-19a	Increased	Recurrence in Colorectal cancer	Matsumura et al. (2015)
ExomiR-3653	Increased	Pancreatic neuroendocrine tumour	Gill et al. (2019)
ExomiR-1307-5p and exomiR-103	Increased	Hepatocellular carcinoma	Fang et al. (2018a), Eun et al. (2020)
ExomiR-1247-3p	Increased	Liver cancer	Fang et al. (2018b)
ExomiR-718	Decreased	Recurrence in hepatocellular carcinoma	Sugimachi et al. (2015)
ExomiR-21	Increased	Esophageal squamous cell cancer	Tanaka et al. (2013)
ExomiR-497-5p	Decreased	Non-small cell lung cancer	Huang et al. (2019)
ExomiR-141, exomiR-146b-3p and exomiR-194	Increased	Recurrence in prostate cancer	Selth et al. (2013)
ExomiR-275	Increased	Recurrence in bone marrow In prostate cancer	Jiang et al. (2022)
ExomiR-148a-3p	Decreased	Lymph node metastasis in ovarian cancer	Gong et al. (2016)
ExomiR-21	Increased	Recurrence in glioma	Shi et al. (2015)
ExomiR‐375	Increased	Bone marrow metastases in patients with neuroblastoma	Colletti et al. (2020)

### 2.1 Gastrointestinal tumors

In a study of colorectal cancer (CRC), a set of exomiRs including exomiR-92 was significantly overexpressed in plasma and tissue samples (Ng et al., 2009). This study has also suggested that exomiR-92 can be used as potential biomarker to detect colorectal cancer since it was differentially expressed in patients with colorectal cancer compared to gastric cancer (Ng et al., 2009). In another study of CRC patients, there were significant increased in exomiR-92a-3p and exomiR-17-5p levels in serum samples and this increase correlates with the stage and grade of the cancer (Fu et al., 2018) which suggest the value of exomiR not only as diagnostic biomarker but as prognostic biomarker. A study on gastric cancer reported significant levels of exomiR-17-5p, exomiR-21, exomiR-106a and exomiR-106b in plasma from patients with gastric cancer compared to healthy individuals (Tsujiura et al., 2010). Expression analysis of exomiR profile including exomiR-21, exomiR-155, exomiR-210 and exomiR-196a isolated from plasma is shown to be associated with pancreatic adenocarcinoma patients (Wang et al., 2009). ExomiR-210 from plasma was also altered in two independent cohorts with pancreatic cancer (Ho et al., 2010). Increase expression of exomiR-500 was observed in serum of patients with hepatocellular carcinoma (HCC) (Yamamoto et al., 2009). Expression levels of exomiR-184 in plasma were significantly higher in patients with squamous-cell carcinoma of the tongue compared to healthy individuals and its levels decreased significantly after tumour removal (Wong et al., 2008). Although the majority of the studies focused on circulating exomiRs in serum and plasma, further studies have investigated the potential use of exomiRs as diagnostic/prognostic biomarker for cancer in other body fluids. For example, expression levels of exomiR-125a and exomiR-200a in saliva from patients with oral squamous-cell carcinoma were significantly reduced compared to healthy individuals (Park et al., 2009).

**FIGURE 2:**
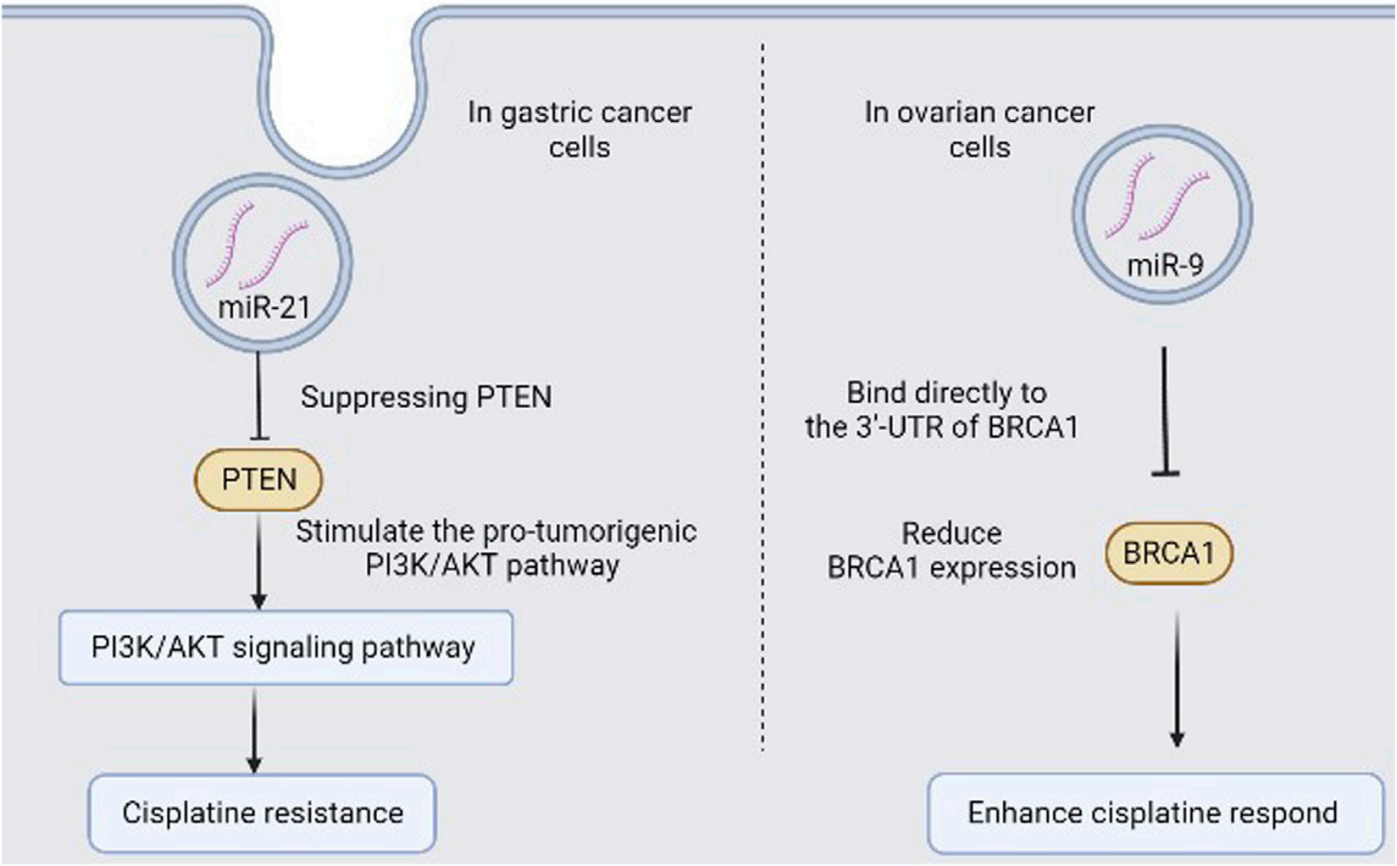
Schematic for two examples of exomiRs in drug response/drug resistance in cancer.

Chemotherapy is one of the most common therapeutic approaches to treat cancer. Successful response to initial therapy is often dependent on type of treatment and tumour type and can be identified by disease progression while resistance to drugs often accompanies with recurrence of tumour. The mechanisms of how cancer can resist treatment have been previously identified (Zahreddine and Borden, 2013) and exomiRs mediating intercellular communication have been identified as one of the mechanisms (Maacha et al., 2019). Tumour cells in their microenvironment can exchange genetic materials and mediate intracellular communications through secreted exomiRs which can promote tumour progression (Lin and Gregory, 2015). In CRC study, exomiR-181b was over-expressed in tumour biopsy compared to normal tissues and was associated with response to 5-fluorouracil treatment (Nakajima et al., 2006). Colon cancer cells and human osteosarcoma cells with elevated levels of exomiR-140 and exomiR-215 have been shown resistance to methotrexate, 5-fluorouacil, and Tomudex (Song et al., 2009; Song et al., 2010). FOLFOX chemotherapy is usually giving to patients with advanced CRC as first-line treatment, and half of the patients acquire resistance with no reliable approach to predict resistance. ExomiR-19a was noticed to be upregulated in patients serum with FOLFOX-resistance and further analysis showed that exomiR-19a can predict acquired drug resistance (Chen et al., 2013) which suggest the use of serum exomiR-19a as a potential biomarker to predict resistance in FOLFOX for advanced CRC patients. Gemcitabine (GEM), a common chemotherapy drug used to treat cancer patients, with promising results, but cancer patients often develop resistance after going long-term treatment. Alternation in specific exomiRs level may play a role since long-term administration with GEM has been shown to associate with increase exomiR-155 level which mediate anti-apoptotic activity that led to chemoresistance in pancreatic ductal adenocarcinoma (Mikamori et al., 2017). Therefore, exomiR-155 could predict GEM-resistance and could be used as novel therapeutic target for GEM treatment in pancreatic ductal adenocarcinoma, based on its function as a driver of resistance. Docetaxel can induce tumour cell apoptosis through Bcl-2 which can be regulated by exomiR-34a (Corcoran et al., 2014). Increasing exomiR-34a level can reduce cell viability and enhance hepatocellular carcinoma cell lines sensitivity to sorafenib through reduced Bcl-2 expression (Yang et al., 2014). When exomiRs internalized through endocytosis in tumour cells, exomiRs can regulate their response to cell signals (Hannafon and Ding, 2013) and inhibit cell apoptosis by suppressing PTEN and stimulate the pro-tumorigenic PI3K/AKT pathway (Zheng et al., 2017). Such a process can promote cisplatin resistance (Radisavljevic, 2013) (Figure 2). In addition, elevated level of exomiR-122 in patients could predict better response to taxol as suppressing exomiR-122 can lead to increase septin-9 in liver cancer which correlates with resistance to taxol (Sun et al., 2016). Hence, exomiRs can mediate communication to tumour cells, tumour progression and resistance to drugs (Table 2).

Metastasis can result in tumour recurrence and plays a key role in reducing survival rate (Gandaglia et al., 2015). Currently, there are no approaches to predict recurrence of tumour at any stage of the disease. Several exomiRs shave been shown to associate with different stages of cancer (Calin and Croce, 2006), therefore, they could be used as potential biomarkers to predict recurrence of tumour (Table 3). Increase levels of exomiR-106a-5p in gastric cancer patients is correlated with the potential to promote metastases (Yuan et al., 2016). In colon cancer patients diagnose at stage II and III, exomiR-4772-3p levels have been associated with elevated risk of tumour recurrence and reduced overall survival (Liu et al., 2016a), and a increased expression of exomiR-19a has been associated with tumour recurrence in colorectal cancer (Matsumura et al., 2015). In pancreatic neuroendocrine tumour study, upregulation of exomiR-3653 was associated with high risk of tumour recurrence through interaction with ATRX (Gill et al., 2019). Circulating exomiR-1307-5p was reported to promote tumour metastasis in HCC through promoting epithelial–mesenchymal transition (EMT) (Eun et al., 2020). ExomiR-103 can reduce the integrity of endothelial cell junction and promote the permeability of vessels through targeting of VE-Cadherin and p120-catenin and ZO-1 which results in transendothelial infiltration of HCC cells and promote metastasis (Fang et al., 2018a). Furthermore, increased expression levels of exomiR-1247-3p is associated with lung metastasis in patients with liver cancer (Fang et al., 2018b). HCC recurrence is important factor of therapy such as liver transplantation. Screening exomiR-718 levels in HCC can predict poor prognosis after liver transplantation and HCC recurrence (Sugimachi et al., 2015). Furthermore, in esophageal squamous cell cancer patients, elevated exomiR-21 level is correlated with metastasis (Tanaka et al., 2013).

### 2.2 Lung cancer

A study examined exomiR-expression profile from serum and found 63 exomiRs to be associated with non-small-cell lung cancer (NSCLC) (Chen et al., 2008a). This study showed that the level of these exomiRs profile differed from serum and blood cells from NSCLC patients while it was the same in healthy individual which suggests that tumour-specific exomiRs in serum were derived from cancer cells. In addition, another board of 34 exomiRs were found in asymptomatic NSCLC patients serum (Bianchi et al., 2011). These reports suggest potential use of exomiRs as non-invasive surrogate diagnostic markers for cancer, potentially of value in screening of asymptomatic populations. A study generated exomiR profile consist of 21 exomiRs analysed from plasma samples collected 12–28 months before lung cancer diagnosis and at the time of detection which suggest a potential diagnosis and prognosis biomarkers for lung cancer (Boeri et al., 2011). Further exomiRs analysis was tested on tissues biopsy took from lung adenocarcinoma patients shows increased levels in panel containing 12 exomiRs including hsa-miR-212, -214, −205, −210, −203, −191, −192, −146, −155, −21, −106a and -17-3p (Rabinowits et al., 2009). A following study used the same approach in screening exomiRs and found similar exomiRs profile in both the tissue biopsy and plasma-derived exosomes from lung adenocarcinoma patients with elevated level of exomiR-200-5p, exomiR-379, exomiR-139-5p and exomiR-378a which suggest a useful biomarker for lung adenocarcinoma (Cazzoli et al., 2013). Loss of exomiR-128b, an EGFR regulator, was associated with better response to gefitinib, an EGFR inhibitor, in patients with relapsed NSCLC (Weiss et al., 2008) and high expression levels of exomiR-21 were found in patients with vertebral column metastasis through increased expression of COX-19 (Guo et al., 2015). NSCLC patients with reduced level of exomiR-497-5p have high chance of metastases (Huang et al., 2019). ExomiR-497-5p can regulate multiple mRNAs including FGF2-encoding mRNAs which can result in migration and invasion (Huang et al., 2019).

### 2.3 Prostate cancer

A study shows the expression levels of exomiR-141 detected in serum were used to distinguish prostate cancer patients from heathy individuals (Lodes et al., 2009), with other 15 exomiRs including exomiR-16 and exomiR-92a/b were highly expressed in prostate cancer patients (Lodes et al., 2009). In addition, increase levels of exomiR-21-5p, exomiR-141-5p and exomiR-574-3p were also observed in urine samples from patients with prostate cancer (Samsonov et al., 2016). Furthermore, serum exomiR-21 expression levels were associated with resistant to docetaxel-based chemotherapy compared to patients with chemosensitive response in prostate cancer (Zhang et al., 2011a). Prostate cancer patients with increase levels of exomiR-194, exomiR-146b-3p and exomiR-141 have high chance of poor prognosis and recurrence (Selth et al., 2013). Bone metastasis is common in patients with prostate cancer and mediate disease complication (Kfoury et al., 2021). ExomiR-275 derived from prostate cancer has been reported to mediate bone metastasis in prostate cancer patients (Jiang et al., 2022).

### 2.4 Ovarian and breast cancer

A study on epithelial ovarian cancer found eight exomiRs from serum including exomiR-92, exomiR-93 and exomiR-126 were highly expressed in 19 patients compared to 11 healthy individuals (Resnick et al., 2009). A panel of exomiRs containing exomiR-205, exomiR-214, exomiR-200b, exomiR-203, exomiR-200a, exomiR-200c, exomiR-21 and exomiR-141 were noticed to be escalated in exosomes isolated from patients serum with ovarian cancer (Taylor and Gercel-Taylor, 2008). A prospective analysis showed significant alternation in exomiR-195 in serum, plasma, or whole blood collected from patients with breast cancer (BC) (Heneghan et al., 2010). The serum levels of exomiR-195 was remarkably decreased after tumour removal (Heneghan et al., 2010). Furthermore, BC patents have elevated level of serum exomiR-101 and exomiR-372 compared to healthy controls (Eichelser et al., 2014) and patients with triple-negative BC have increased level of exomiR-373 increased level of exomiR-373 (Eichelser et al., 2014). This suggests, the use of these exomiRs as potential diagnostic markers for BC.

Increase level of certain exomiRs can enhance sensitivity of many chemotherapy drugs. For instance, exomiR-9 downregulates BRCA1 protein through direct binding to the 3′-UTR of BRCA1 mRNA and reduce the ability of the BRCA complex to repair damage in DNA. Therefore, increase exomiR-9 can suppress DNA damage repair in ovarian cancer and enhance ovarian cancer respond to chemotherapy, such as cisplatin (Figure 2) (Sun et al., 2013). BC patients with genetically deleted chromosome 11q which containing the *miR‐125 b* gene often show better respond to chemotherapy with anthracycline (Climent et al., 2007) which suggests a potential association between exomiR-125b dysregulation and response to drugs containing anthracycline in BC patients. Reduced levels of exomiR-148a-3p have been shown to increase chance of tumour metastasis in ovarian cancer (Gong et al., 2016) which suggest the use of exomiR-148a as a potential marker for tumour recurrence and, possibly, tumour invasion and migration in ovarian cancer.

### 2.5 Neuroblastoma

Patients with recurrent glioma have higher cerebrospinal fluid exomiR-21 levels compared to non-tumour control group, however, no differences were observed in exomiR-21 isolated from serum (Shi et al., 2015). ExomiR‐375 has been reported to promote bone marrow metastases in patients with neuroblastoma (NB) (Colletti et al., 2020) by downregulating YAP1 levels which enhance osteogenic differentiation of mesenchymal stromal cells (Colletti et al., 2020). Additional screening of exomiRs in metastatic sites and primary tumour sites is important to enhance prediction of recurrence and metastasis.

First-line treatment for glioma patients is usually temozolomide (TMZ) (Chibbaro et al., 2004), however, there is no reliable approach to predict which patients will be resistance to TMZ. Interestingly, downregulation of exomiR-195, exomiR-455-3p, and exomiR-10a has been associated with acquired TMZ-resistance (Ujifuku et al., 2010). Furthermore, TMZ resistance in glioma cells have been reported to be associated with dysregulation with exomiR-221 level (Yang et al., 2017a). These studies, recommend screening for exomiR-10a, exomiR-122, exomiR-455-3p and exomiR-195 levels before and during TMZ therapy to identify better treatment approach for the patient.

### 2.6 Pediatric tumours

The non-invasive diagnosis, limited-risk and availability of the exomiRs in the body fluids make them attractive method to diagnose cancer in pediatric patients (Galardi et al., 2019). NB is one of the most common tumour in children with heterogeneous clinical characteristics (Ma et al., 2019). Profile for exomiRs isolated from 17 NB patients plasma have been shown different expression compared to healthy controls (Ma et al., 2019). The significant expression of exomiR-199a-3p was correlated with severity of NB patients (Ma et al., 2019), as it increases proliferation and migration of NB cells *in vitro* (Ma et al., 2019). In another study, high expression levels of exomiR-16 were linked to poor prognosis in childhood acute lymphoblastic leukemia (ALL) (Kaddar et al., 2009).

Another pediatric tumour accounts for 80% of primary tumour liver in young children infant is Hepatoblastoma (HB) (Ranganathan et al., 2020). The expression of exomiR-21 in plasma was higher in HB patients compared to healthy control (Liu et al., 2016b), which makes it a good diagnosis and prognosis biomarker for HB. Further analysis studies of exomiRs profile have shown increased expression of three-miRNA-based expression signature (exomiR-7112-5p, exomiR-885-3p and exomiR-1245a) in plasma of acute myeloid leukaemia patients (AML) (Zampini et al., 2017), increased expression of exomiR-25-3p in serum of osteosarcoma patients (Fujiwara et al., 2017) and decreased expression of exomiR-125b in serum of ewing’s sarcoma patients (Nie et al., 2015). These results showed evidence of using exomiRs as a potential diagnostic and prognostic biomarkers for pediatric tumours. In pediatric AML, both exomiR-29a and exomiR-100 were indicator for better response to chemotherapy (Said et al., 2022). Increased expression levels of exomiR-125b were associated with drug resistance in pediatric acute promyelocytic leukemia (Zhang et al., 2011b).

### 2.7 Hematologic tumours

Alternation in exomiRs expression is potential biomarker not only in solid tumours, but also in non-solid hematologic tumours. For example, increase expression of exomiR-21, exomiR-155, and exomiR-210 was discovered in the serum of diffuse large B cell lymphoma (DLBCL) patients (Lawrie et al., 2008). Moreover, there was a significant decrease of exomiR-92a in plasma collected from patients with acute leukemias compared to healthy individuals (Tanaka et al., 2009). In addition, plasma samples collected from multiple myeloma (MM) patients have elevated levels of exomiR-148a, exomiR-181a, exomiR-20a, exomiR-221, exomiR-625, and exomiR-99b compared to healthy individuals (Huang et al., 2012a). The increased levels of both exomiR-20a and exomiR148a were associated with short relapse-free survival rate (Huang et al., 2012a). The expression profile of exomiR-128a, exomiR-128b, let-7b, exomiR-223 can help distinguish ALL from AML with over 95% accuracy rate (Mi et al., 2007). Alternation expression of exomiR-32, exomiR-98 and exomiR-374 in blood samples is observed in chronic lymphocytic leukemia (CLL) patients (Rahimi et al., 2021). In a chronic myelogenous leukemia (CML) studies, increased levels of exomiR-451 was observed in plasma samples from CML patients in the chronic stage (Keramati et al., 2021) while increased levels of exomiR-126, exomiR-155, and exomiR-222 were observed during blast crisis (Machová Poláková et al., 2011).

Increased expression levels of exomiR-142-3p and the exomiR-17-92 were associated with glucocorticoid-resistant B cell precursor acute lymphoblastic leukemia (Sakurai et al., 2019). Furthermore, resistance to daunorubicin and vincristine in pediatric ALL was associated with increased expression levels of exomiR-99a, exomiR-100, and exomiR-125b (Schotte et al., 2011).

In CML, tyrosine kinase inhibitors (TKIs) resistance was associated with decreased expression levels of exomiR-142-5p, exomiR-199b, exomiR-217, exomiR-221, and exomiR-365a-3p (Yeh et al., 2016; Jiang et al., 2018; Klümper et al., 2020). In DLBCL studies, upregulation of exomiR-155 and reduced expression levels of exomiR-193b-5p and exomiR-1244 were linked to treatment failure with rituximab plus doxorubicin, cyclophosphamide, prednisone, and vincristine (Iqbal et al., 2015; Bento et al., 2022). Reduced expression levels of both exomiR-145-3p and exomiR-155 were associated with bortezomib resistance in MM (Amodio et al., 2019; Wu et al., 2020). ExomiR-217 sensitizes AML to doxorubicin *via* targeting KRAS (Xiao et al., 2017) while exomiR-143 enhances chemosensitivity of AML to cytosine arabinoside by targeting ATG7-and ATG2B-dependent autophagy (Zhang et al., 2020a). In CLL, fludarabine response was associated with increased expression levels of exomiR-181a/b (Zhu et al., 2012). Another study has shown a role of exomiR-181a in GC resistance in MM cells (Huang et al., 2012b). Elevated expression of exomiR-485-3p increased sensitivity to Top2 inhibitors in CEM/VM-1-5 cells through regulating the NF-YB expression level (Chen et al., 2011). Downregulation of exomiR-451 was associated with Imatinib resistance in CML (Scholl et al., 2012). In patients with refractory AML, downregulation of exomiR-let-7f was associated with Adriamycin resistance (Dai et al., 2014). Plasma levels of let-7a and exomiR-16 were significantly decreased in patients with myelodysplastic syndrome which predict with both progression-free survival and overall survival (Zuo et al., 2011).

## 3 ExomiRs as immunotherapeutic targets to regulate immune checkpoint molecules

The therapeutic potential of Immune-checkpoint blockade in cancer has improved the overall survival in the last years (Postow et al., 2015). The first checkpoint blockade to receive FDA approval was ipilimumab, which is an antibody that target cytotoxic T-lymphocyte-associated protein 4 (CTLA4) (Hodi et al., 2010). FDA has also approved additional two immune checkpoint blockade antibodies that target programmed cell death protein 1 (PD-1) known as pembrolizumab and nivolumab to treat stage IV melanoma (Robert et al., 2015a; Robert et al., 2015b) and NSCLC (Brahmer et al., 2015; Garon et al., 2015). Although these targeting antibodies have shown promising results in cancer treatment, the systematic administration of these protein-format and murine-origin blocking antibodies can result in undesirable immune-related adverse events (irAEs) (Van Hoecke and Roose, 2019). ExomiRs secreted from cancer cells can regulate stromal cells and promote cancer angiogenesis (Wu et al., 2019). In addition, they play a key role in intercellular transmission of signals that regulate immune checkpoint molecules and influence the function of several immune cells such as dendritic cells and T cells which are important cells in cancer immunotherapy (Huemer et al., 2021). Several exomiRs can regulate the expressions of immune checkpoint molecules, mimicking the therapeutic impact of immune checkpoint blocking antibodies and controlling the irAEs associated with the administration of the blocking antibodies (Van Hoecke and Roose, 2019). Therefore, they have a potential role as immunotherapeutic agents to regulate immune checkpoint molecules expression either as exomiR mimics or exomiR antagonists. The exomiR mimics function to restore the tumour suppressor capabilities of exomiR while the exomiR antagonists serve as inhibitors (Bader et al., 2011).

The first exomiR mimic to enter the clinical trial phase was the use of MRX34, an exomiR-34a mimic (Bader, 2012; Austin, 2013). In AML, exomiR-34a was found to target PD-L1 mRNA and downregulate PD-L1 expression (Wang et al., 2015). In addition, combination therapy of radiotherapy (XRT) and administration of MRX34 result in increased CD8^+^ T cell infiltration and reduced Treg in NSCLC (Cortez et al., 2016). These results suggest a potential role of exomiR-34a mimic to enhance anti-tumour immunity and reduce tumour growth. Further studies have examined new potential targets for exomiR mimics. For example, exomiR-424 has the ability to supress PD-L1 and CD80 expression, and restores cytotoxic CD8^+^T cells effector function and improves the survival in ovarian carcinoma mouse model (Xu et al., 2016). The exomiR inhibitors have also shown immunotherapeutic potential. Cobomarsen (MRG105) is an anti-exomiR-155 agent has shown reduce tumour growth when administrated systematically (Van Roosbroeck et al., 2017). Another study of melanoma, adoptive transfer of CD8^+^ cytotoxic T lymphocytes treated with exomiR-23a inhibitor has shown decreased in tumour growth and increased effector function of CD8^+^ cytotoxic T lymphocytes (Lin et al., 2014). Furthermore, exomiR-149-3p reduces inhibitory receptors and revised CD8^+^ T cell exhaustion in breast cancer cells (Zhang et al., 2019), and exomiR-5119 enhance BC immunotherapy through regulation of immune checkpoints in dendritic cells (Zhang et al., 2020b). In addition, exomiR-34a-5p can regulate the expression of PDL-1 in AML (Wang et al., 2015). ExomiR-138-5p can regulate PDL-1 expression in colorectal cancer (Zhao et al., 2016) and regulate PD-1 expression in glioma (Wei et al., 2016). Low expression levels of exomiR-200 in NSCLC cells are associated with increased expression levels of PD-L1 since exomiR-200 have been shown to target 3′UTR of PD-L1 and decreases its expression (Chen et al., 2014). Therefore, NSCLC patients with exomiR-200 low pattern expression may benefit from the use of miR-200 mimics. Furthermore, decrease expression levels of exomiR-197 are associated with increased expression levels of PD-L1 and promote drug resistance and reduced overall survival in patients with NSCLC (Fujita et al., 2015). Thus, treatment with exomiR-197 mimics may benefit patients with PD-L1-positive NSCLC.

CTLA-4 is another important immune checkpoint molecule which can bind to either CD80 or CD86 on antigen presenting cells and result in suppression the effector function of T cell (Teft et al., 2006). CTLA-4 can be regulated by exomiR-138-5p (Wei et al., 2016). *In vivo* treatment of exomiR-138 mimic results in downregulation of CTLA-4, PD-1 and Foxp3 on tumor infiltrating CD4+T cells leading to significant decrease of T reg in glioma mouse model (Wei et al., 2016). In addition, exomiR-424 reduced CD80 expression in dendritic cells and results in CD80/CTLA-4 blockade and increased T cell activity (Xu et al., 2016). Furthermore, T cell immunoglobulin and mucin-domain containing-3 (TIM-3) is expressed on activated T cell and reduces T cell activity by inducing T cell exhaustion and tolerance (Ferris et al., 2014). In glioma, exomiR-15a/16 knock-out Mice have decreased TIM-3 and PD-1 expression and increased cytokines secretion in tumor-infiltrating CD8^+^ T cells which result in better overall mice survival (Yang et al., 2017b). These examples show a potential role of targeting exomiRs as immunotherapeutic agents to regulate immune checkpoint molecules and enhance anti-tumour immunity.

## 4 Conclusion remarks

Treatment to cancer has improved significantly over the past few decades. However, many patients do not benefit from the treatment due to late-stage diagnosis, tumour recurrence and sometime treatment resistance. Therefore, early diagnosis of cancer and reliable biomarkers for cancer recurrence and treatment resistance are critical to determine the best therapeutic approach and to improve overall survival. To develop and identify a sensitive, and less invasive biomarkers for cancer implication, exomiRs should not be ignored. These circulating exomiRs have the potential for new cancer biomarkers due to several characteristic factors. First, oncogenic pathways can be regulated by exomiRs (Sumazin et al., 2011) which can result in tumour development, suppression and can mediate treatment response which makes it good candidate for cancer progression biomarker. The unique expression profiles of exomiRs in tumours helps in providing wide range of information for tumour stage, recurrence and treatment resistance which makes it a good candidate for liquid biopsy without the need for tissue biopsy. The lipid biolayer structure in the exosomal membrane protect exomiRs from degranulation in the biofluid; this protection makes it more desirable compared to other molecules which can be degraded in harsh conditions like extreme temperature and prolong storage (Chen et al., 2008b). The easy accessibility is another reason for their desirable use, exomiRs can be obtained in less-invasive way from liquid biopsy including blood, urine and saliva. The change in exomiRs expression profile observed in these bio-fluid can be seen as early as early stage of tumour (Jiménez-Avalos et al., 2021).

Based on the advantages of exomiRs derived from tumour, it can be used as a potential biomarker for cancer implication as it can predict the growth, spread, recurrence and treatment-resistance of tumours. However, several studies reported unsuccessful validation in using exomiRs as biomarkers for cancer (Sapre et al., 2014; Wan et al., 2014). This could be due to several factors including limited methodologies to access and obtain exomiRs and time of collection and the cancer stage (Mateescu et al., 2017). These studies highlight the significance of incorporating methodological approaches to obtain exomiRs and to ensure the reproducibility of the result in future studies.

The discovery of immune checkpoint blockade as cancer immunotherapy has improved the overall survival significantly, however, new strategies on how to regulate their expression using something other than protein-format and murine-origin antibodies are crucial to avoid undesirable irAE. ExomiRs play a key role in posttranscriptional control of protein expression and therefore, may be of better target since some exomiRs expression levels are associated with expression of immune checkpoint molecules. Further investigations are important to deeply show the effect of exomiRs on cancer biology.

## 5 Future direction

The variability of cancer lends itself in the growing field of personalized medicine which provide great patient benefit (Verma, 2012). Artificial intelligence (AI) technique is emerging in personalized medicine and biomedical research which include cancer clinical implications including cancer diagnosis, treatment, and the discovery of new potential therapy (Schork, 2019; Elemento et al., 2021). The big data obtained from thousands of studies related to exomiRs in cancer can leverage an opportunity to implement AI in cancer clinical implication and improve cancer diagnosis (Paolini et al., 2022). Together, these computational platforms can provide a new technique in cancer clinical implication and provide a modern approach on the validity of exomiRs signature as biomarker in cancer and immunotherapeutic agents.
